# Feasibility and Safety of the C1 “Zero Angle” Screw: A Novel “In–Out–In” Technique for Atlantoaxial Dislocation

**DOI:** 10.1111/os.14309

**Published:** 2024-12-03

**Authors:** Zexing Chen, Xinzhao Huang, Xiaobao Zou, Peirong Lian, Guoqiang Liu, Junlin Chen, Changrong Zhu, Xiangyang Ma

**Affiliations:** ^1^ The First School of Clinical Medicine Southern Medical University Guangzhou China; ^2^ Department of Orthopedics General Hospital of Southern Theatre Command of PLA Guangzhou China; ^3^ Guangzhou University of Chinese Medicine Guangzhou China; ^4^ Department of Spinal Surgery, Orthopedic Medical Center, Zhujiang Hospital Southern Medical University Guangzhou China

**Keywords:** atlantoaxial dislocation, atlas, in–out–in, narrow pedicle, zero angle

## Abstract

**Objectives:**

To minimize the risk of V3 segment of vertebral artery (VA) injury in the atlantoaxial dislocation (AAD) patients with C1 pedicle height less than 4.0 mm and provide a strong toggle force in irreducible AAD and revision surgery. We evaluated the feasibility of C1 “Zero Angle” screw (C1ZAS) and safe entry point with “in–out–in” technique as an alternative option for C1 pedicle screw (PS) in cases with AAD.

**Methods:**

Sixty‐one patients with AAD or atlantoaxial instability (AAI) (45 male and 16 female) who underwent cervical computed tomography and magnetic resonance imaging scans in our center between January 1, 2022 and December 31, 2023 were retrospectively reviewed. Measurements were made around the ideal trajectory and entry point of C1ZAS using computerized tomography (CT) and magnetic resonance imaging (MRI) in 61 patients. Radiographic measurements included (A) the distance from the recess to the transverse foramen (RTF); (B) the tricortical screw zone (TSZ); (C) the lateral mass height along the C1ZAS trajectory (LMH); (D) the screw length of C1ZAS (ZSL); (E) the screw length of C1 PS (PSL); (F) the distances from the recess to the dura (RD); (G) the distance from the recess to the spinal cord (RSC); (H) the distance from the inner of lateral mass to the spinal cord (ILMSC). During the period of January 1, 2022 to December 31, 2023, C1ZAS placement with “in–out–in” technique was used as an alternative option for C1 PS in 8 patients with AAD and unilateral/bilateral narrow C1 pedicles.

**Results:**

The average RTF, TSZ, LMH, ZSL, RD, RSC, and ILMSC were 7.71, 6.14, 8.32, 33.23, 4.68, 10.02, and 2.91 mm respectively. The entry point of the C1ZAS was defined as the projection point of the inner of the recess to the posterior arch and the trajectory should be angled cephalad by 8.7° and medially by 0°. The 61 patients (122 sides) with AAD or AAI were classified into three groups: the low‐risk (76 sides, 62%), the intermedial‐risk (18 sides, 15%), and the high‐risk (28 sides, 23%) groups. Satisfactory C1ZAS placement and atlantoaxial reduction were achieved in all eight patients with AAD and unilateral/bilateral narrow C1 pedicles. No instance of C1ZAS placement‐related VA injury or dural laceration was observed.

**Conclusions:**

When the placement of C1 PS is not feasible in patients with AAD and unilateral/bilateral narrow C1 pedicles, C1ZAS placement with “in–out–in” technique can be considered an effective alternative option, providing tricortical or quadricortical purchase for rigid fixation of the atlas.

## Graphical abstract

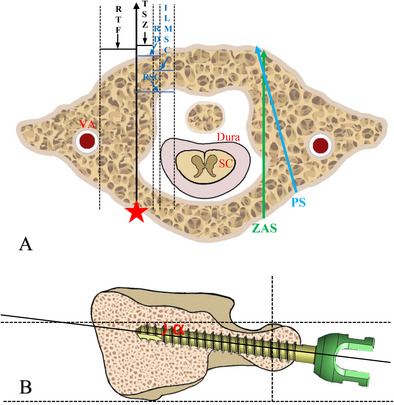

## 1 Introduction

The atlantoaxial region is located at the craniocervical junction, and is connected to the lower skull and cervical vertebra [1]. Traumatic fractures, inflammation, congenital malformations, tumors, and degenerative changes can lead to atlantoaxial dislocation (AAD) or atlantoaxial instability (AAI), compressing the spinal cord (SC), and medulla oblongata. This can cause limb numbness and weakness as well as urination and defecation disturbance. AAD often requires surgical treatment to relieve symptoms and prevent progression [2]. C1 pedicle screw (PS) combined with C2 pedicle or pars screw fixation, have become the most popular fixation techniques in AAD [3, 4, 5]. However, morphologic studies have demonstrated that the height of the C1 pedicle restricts the placement of C1 PS. Qian et al. [6] have found that the average extra medullary height of all the atlas pedicles was 4.43 mm and 23.3% of patients had C1 pedicle extra medullary height < 4 mm which indicating that 23.3% of the population was not feasible for C1 PS placement. When the height is < 4.0 mm, the C1 pedicle cannot accommodate 3.5 mm screws and the risk of V3 segment of vertebral artery (VA) injury is inevitable [7]. Any alternative fixation methods (such as C1 lateral mass screws (LMS), posterior arch crossing screws, or laminar hooks) may not provide a strong enough toggle force, potentially leading to intraoperative or postoperative screw loosening, particularly in cases of irreducible AAD and revision surgery.

In the present study, we describe a novel technique of C1 “Zero Angle” screw (C1ZAS) with radiographic parameters and the preliminary clinical application. This technique has a trajectory parallel to the sagittal plane, providing tricortical or quadricortical purchase for rigid fixation of the atlas while minimizing the risk of V3 segment of VA injury. To the best of our knowledge, the use of C1ZAS to improve stability and safety of internal fixation has not been reported previously. This study was designed: (i) to verify the feasibility and safety of C1ZAS via imaging evaluation; (ii) to explore the effective surgical procedure; and (iii) to assess the radiographic and clinical outcomes of the preliminary clinical application of C1ZAS with “in–out–in” technique in this cohort.

## 2 Materials and Methods

### 2.1 Patients' Data

After obtaining Institutional Review Board (IRB) approval (no: NZLLKZ2024132), we selected cervical computed tomography (CT) and magnetic resonance imaging (MRI) scans of 61 patients (45 males and 16 females) performed between January 1, 2022 and December 31, 2023. The patient inclusion criteria were as follows: (i) Patients with AAD or AAI were presented on imaging; (ii) had cervical CT or MRI examination; and (iii) was over 18 years of age. The patient exclusion criteria were as follows: (i) Patients complicated with rotatory AAD, cervical fracture, or upper cervical deformity; and (ii) had previous upper cervical spine surgery. During the period of January 1, 2022 to December 31, 2023, C1ZAS with “in–out–in” technique was used in 8 patients with unilateral/bilateral narrow C1 pedicles for the atlas fixation. In this part, the patient inclusion criteria were as follows: (i) Patients were performed atlantoaxial posterior fusion technique with unilateral/bilateral C1ZAS; and (ii) with intact radiographic and clinical data at pre‐, post‐operation, and the last follow‐up at least 3 months. Patients with incomplete radiographic and clinical data were excluded. The open‐mouth, lateral, and dynamic cervical X‐radiography, CTA with three‐dimensional reconstruction, and MRI were performed in all patients.

### 2.2 Radiological Measurement

**FIGURE 1:**
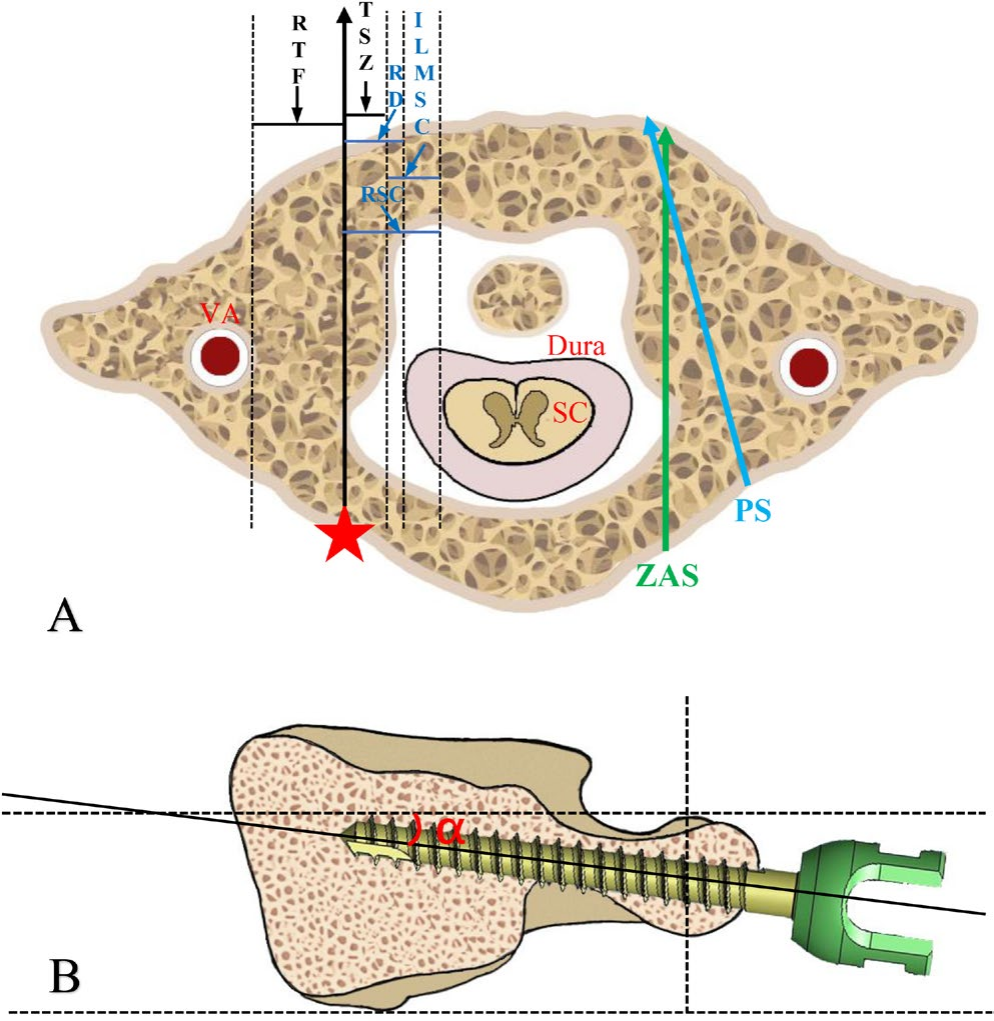
Illustration of the axial plane of the atlas and the parameters of C1ZAS with “in–out–in” technique. (A) The red star was the entry point of C1ZAS and the black solid line was the trajectory of C1ZAS. Black‐dashed lines were parallel lines of the trajectory of C1ZAS. RD, the distance between the recess and the dura. RSC, the distance between the recess and the SC. ILMSC, the distance between the inner of lateral mass and the SC. TSZ, the tricortical screw zone, was measured between the recess and the inner of the lateral mass that could form tricortical fixation. LMH, the lateral mass height along the orientation of the C1ZAS trajectory. The blue solid line and green solid line represented the screw length of conventional PS and C1ZAS. (B) Cephalad angle (α), the angle between the ideal trajectory and the ideal inferior border of C1 vertebral body. The ideal inferior border was defined as the plane perpendicular to the axis of the spinal canal on the C1 vertebrae level.

The C1ZAS with “in–out–in” technique is presented as an alternative to C1 PS in cases of AAD with a trajectory parallel to the sagittal plane. This method involves penetrating the recess of the atlas to achieve tricortical or quadricortical purchase for rigid fixation while minimizing the risk of injury to the V3 segment of the VA. All parameters evaluated in the present study are shown in Figure 1. The distances between the recess and the transverse foramen (RTF), tricortical screw zone (TSZ), lateral mass height (LMH), and screw length (SL) were measured on CT scans. The distances from the recess to the dura (RD), the recess to the spinal cord (RSC), and inner lateral mass to the spinal cord (ILMSC) were measured on MRI scans.

**FIGURE 2:**
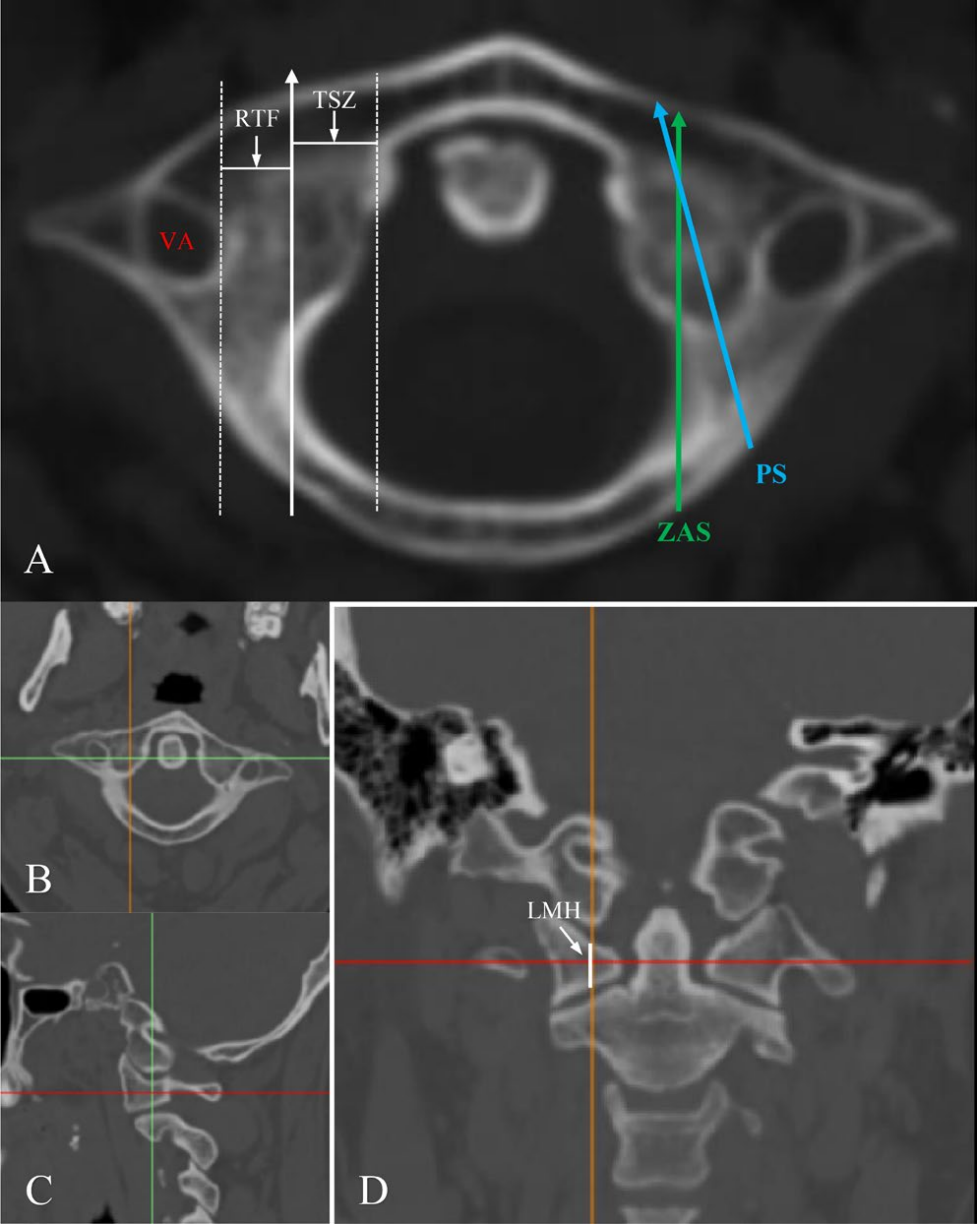
The axial, sagittal and coronal planes of the atlas in CT scans. (A) The white solid line with arrow was the ideal trajectory of C1ZAS. The white‐dashed lines were parallel lines of the trajectory of C1ZAS. RTF was the distance between the recess and the TF. TSZ was the distance between the recess and the inner of lateral mass that could form tricortical fixation. The blue solid line and green solid line represented the screw length of conventional PS and C1ZAS. (B‐D) LMH was measured at the coronal plane (white solid line) along the orientation of the C1ZAS trajectory at the axial and sagittal planes.

CT examinations (Apuilion TSX‐101A, Toshiba, Japan) were performed with a slice thickness of 1.0 mm to 1.5 mm. The CT images were obtained after multiplanar reconstruction on PACS system (Guangzhou, China) and Mimcis research 21.0 (Leuven, Belgium). The parameters were measured at the axial plane of the middle of height and width of the C1 pedicle corresponding to the trajectory of the C1 pedicle axis in the bone window. The RTF was defined as the distance between lines parallel to the sagittal line of the inner of the recess and tangential to the inner wall of the transverse foramen (TF), which is the safe distance from the trajectory to VA. TSZ was measured between the recess and the inner of the lateral mass that could form tricortical fixation without violating the atlantoid joint or ligamentum transversum of the atlas. LMH was measured in the coronal plane along the orientation of the C1ZAS trajectory at the axial and sagittal planes, which allowing for the evaluation of the adequacy of space and safety for C1ZAS placement in the coronal plane. The SL of C1ZAS (ZSL) and conventional C1 PS (PSL) was measured from the entry point to the ventral aspect of the anterior arch of the atlas. The conventional C1 PS was prepared as described by Ma et al. [8] These parameters were visually represented in the axial, sagittal and coronal planes of the CT scans (Figure 2).

**FIGURE 3:**
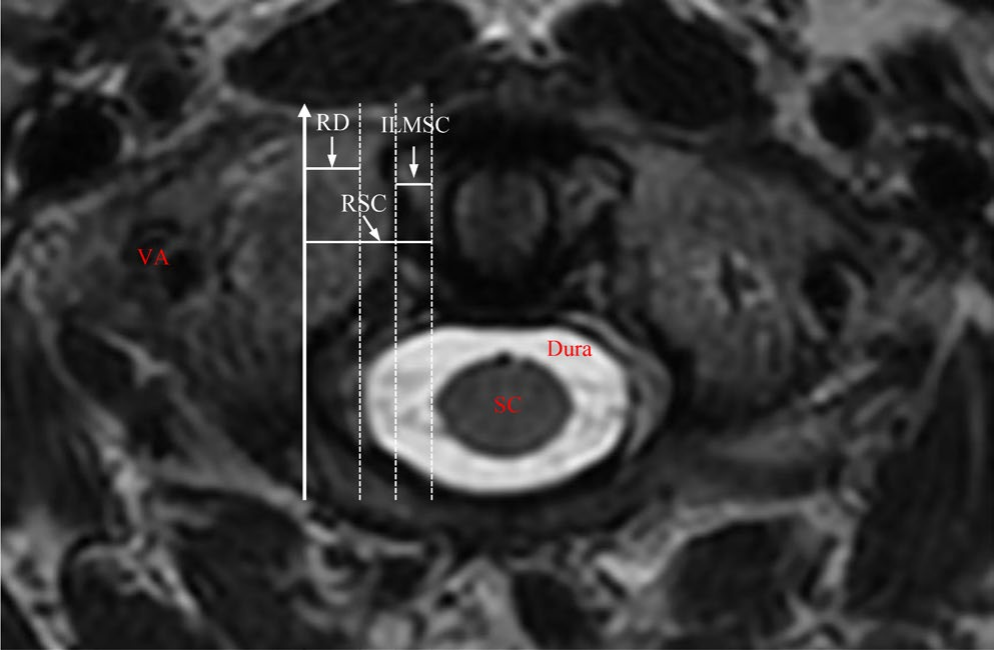
The axial plane of the atlas in MRI scan. The white solid line with arrow was the ideal trajectory of C1ZAS. The white‐dashed lines were parallel lines of the trajectory of C1ZAS. RD was the distance between the recess and dura. RSC was the distance between the recess and the SC. ILMSC was the distance between the inner of the lateral mass and the SC.

MRI scans were performed with a 3T MRI scanner (Signa Hdxt General Electrics, Milwaukee, USA). Images of the axial plane of the atlas were obtained after multiplanar reconstruction on the Mimcis research 21.0. The distance of the following parameters was measured between lines parallel to the sagittal line of the atlas and tangential to the medial/lateral wall of the recess/lateral mass and the dura/SC (Figure 3). RD was defined as the distance from the recess to the dura, representing the safe margin from the trajectory to the dura during C1ZAS placement. RSC was defined as the distance from the recess to the SC, representing the safe margin from the trajectory to SC during C1ZAS placement. ILMSC was defined as the distance from the inner of the lateral mass to the SC, which is used to evaluate the relationship between the TSZ and the safe area for C1ZAS placement.

The commonly used upper cervical screw has a diameter of 3.5 mm; therefore, a diameter of 4 mm is considered the safe threshold for C1ZAS placement. The 61 patients (122 sides) were classified into three groups: low‐risk (RD > 4.0 mm), intermediate‐risk (RD 3.5–4.0 mm), and high‐risk (RD < 3.5 mm) groups.

### 2.3 Surgical Procedure

**FIGURE 4:**
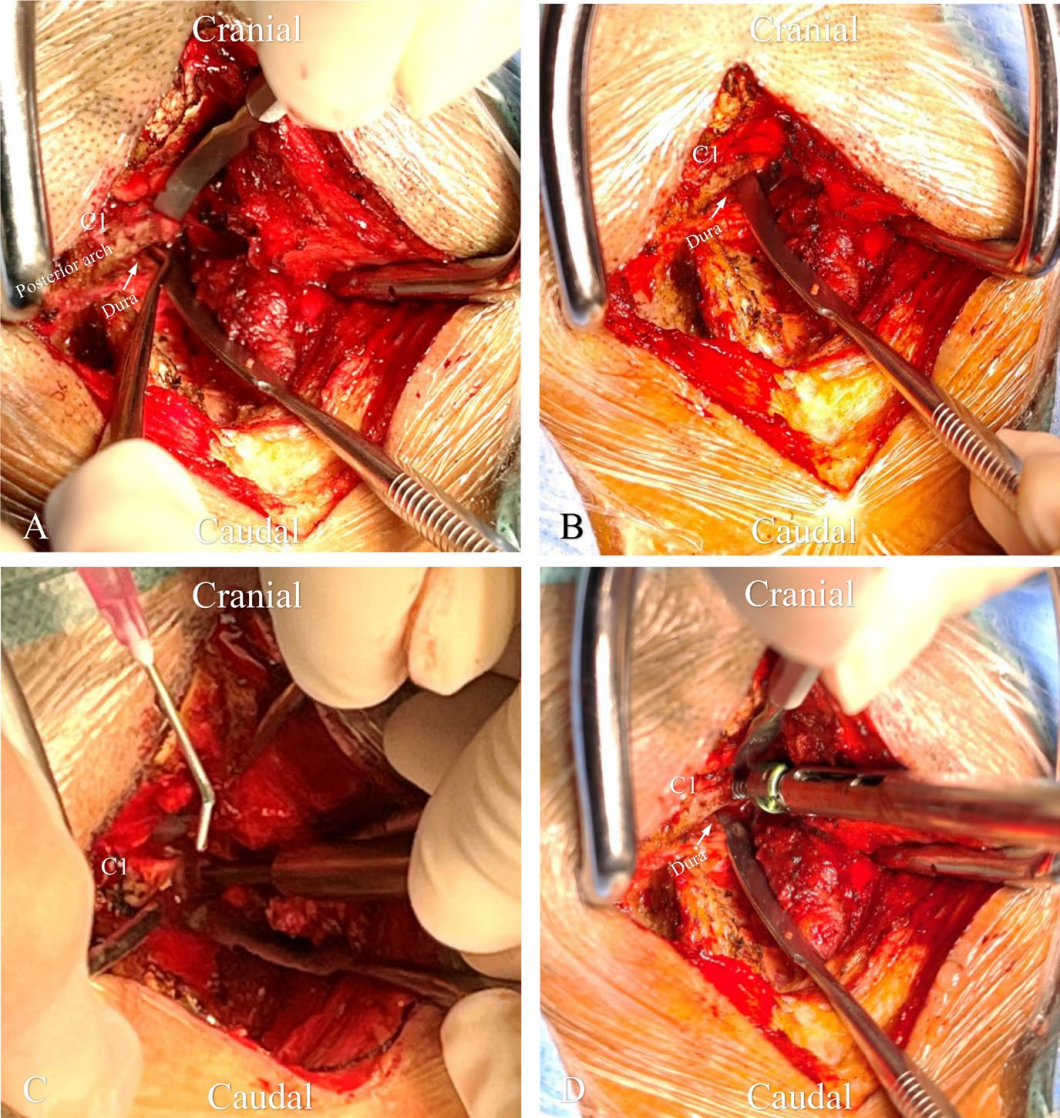
Illustration of C1ZAS placement with “in–out–in” technique and dura protection. (A) A Penfield dissector was used to locate the inner of the recess and extended dorsally perpendicular to the coronal plane to posterior arch at least 3 mm below the superior rim of the C1 lamina. (B) A Penfield dissector should be used slightly retracting the dura by the assistant to protect the dura from injury. (C) A 2.5‐mm power drill and awl were advanced to prepare the screw track along the medial border of the C1 pedicle. Then blunt ball probe was used to verify the integrity of screw track. (D) The screw track penetrated the lateral rim of spinal canal achieving tricortical or quadricortical purchase for rigid fixation of atlas.

All patients underwent skull traction after general anesthesia and posterior atlantoaxial fusion alone with reducible or irreducible AAD. The patients were placed in the prone position. The cervical spine was exposed from the occiput to the C2 spinous process along the posterior midline and to the lateral border of the articulation. A Penfield dissector was used to locate the inner of the recess and extended dorsally, perpendicular to the coronal face, to the posterior arch at least 3 mm below the superior rim of the C1 lamina. The C1ZAS trajectory should be angled cephalad by 8.7° and medially by 0°. The entry point for C1ZAS was decorticated, and then, the pilot hole was prepared using a 2‐mm high‐speed burr. The borders of the C1 pedicle were clearly delineated and the space between the inner cortex of the C1 pedicle and the dura was separated. A Penfield dissector was used, with the assistant slightly retracting the dura to protect it from injury. Subsequently, a 2.5‐mm power drill and awl were advanced to prepare the screw track along the medial border of the C1 pedicle. Then, a blunt ball probe was used to verify the integrity of the screw track. The trajectory was prepared using an appropriately sized tap and a polyaxial screw was inserted. The procedure for screw placement is shown in Figure 4. After screw placement, a piece of gelatin sponge was placed between the screw and the dura. The rods were then contoured and loaded into the C1 and C2 screw heads bilaterally and locked to improve the reduction ulteriorly via the tensile force of the screw‐rod construction. The reduction was repeated until it appeared satisfactory on intraoperative x‐rays or O‐arm navigation. The bone graft bed was prepared by grinding off the C1 posterior arch and C2 lamina with a high‐speed burr. Autografts were harvested from iliac cancellous bone. After applying negative pressure to the drainage tube, the incision was closed layer by layer.

### 2.4 Statistical Analysis

The data were statistically analyzed with independent and paired *t*‐test using SPSS version 24.0 software (IBM Corp., Armonk, NY, USA). All data were normally distributed based on the results of the K–S test, and were expressed as mean ± SD. All parameters were compared for male and female, left and right sides, and the SL of C1ZAS and conventional PS. Then, the intraclass correlation coefficient (ICC) was used to assess the inter‐observer reliability of these parameters. A value of *p <* 0.05 was considered statistically significant.

## 3 Results

### 3.1 General Data

All data of the parameters evaluated in this study are given in Table 1. In total, 61 patients (45 males and 16 females) were involved in this study. No variation in dura, SC, VA, or atlas was found. Furthermore, no significant differences were found in the parameters measured in this study between the left and right sides (*p* > 0.05).

**TABLE 1:** Anatomic parameters of the atlas in AAD patients.

		Total		Male		Female		*t*	*p*
		Mean	±SD	Mean	±SD	Mean	±SD		
RTF (mm)	Right	7.56	1.32	7.65	1.38	7.28	1.10		
	Left	7.87	1.51	7.96	1.49	7.62	1.60		
	Total	7.71	1.42	7.81	1.43	7.45	1.36	1.242	0.217
	*t*	1.691		1.382		0.964			
	*p*	0.096		0.174		0.350			
RD (mm)	Right	4.49	1.50	4.62	1.49	4.11	1.49		
	Left	4.87	1.33	4.93	1.49	4.69	0.75		
	Total	4.68	1.42	4.78	1.49	4.40	1.20	1.292	0.199
	*t*	1.923		1.258		1.930			
	*p*	0.059		0.215		0.073			
RSC (mm)	Right	9.87	1.90	9.83	2.04	9.99	1.54		
	Left	10.17	1.76	10.13	1.98	10.32	0.88		
	Total	10.02	1.83	9.98	2.01	10.16	1.25	−0.588	0.558
	*t*	1.668		1.435		0.912			
	*p*	0.101		0.158		0.376			
ILMSC (mm)	Right	2.72	1.27	2.75	1.35	2.63	1.07		
	Left	3.11	1.23	2.99	1.25	3.44	1.18		
	Total	2.91	1.26	2.87	1.28	3.03	1.18	−0.603	0.548
	*t*	1.765		0.917		2.020			
	*p*	0.083		0.364		0.062			
TSZ (mm)	Right	6.29	1.33	6.47	1.49	5.76	0.48		
	Left	6.00	1.27	6.13	1.29	5.63	1.20		
	Total	6.14	1.31	6.31	1.54	5.62	1.00	2.777	0.007
	*t*	−1.822		−1.821		−0.454			
	*p*	0.073		0.075		0.656			
ZSL (mm)	Right	33.21	2.52	33.72	2.57	31.77	1.74		
	Left	33.25	2.30	33.79	2.13	31.75	2.17		
	Total	33.23	2.40	33.75	2.34	31.76	1.93	4.310	0.000
	*t*	0.203		0.249		−0.094			
	*p*	0.840		0.805		0.926			
PSL (mm)	Right	26.69	1.98	26.84	2.09	26.28	1.63		
	Left	26.85	1.73	27.06	1.77	26.26	1.50		
	Total	26.77	1.85	26.95	1.93	26.27	1.54	1.804	0.074
	*t*	1.039		1.299		−0.080			
	*p*	0.303		0.201		0.937			
LMH (mm)	Right	8.36	1.95	8.38	2.00	8.31	1.85		
	Left	8.30	1.71	8.43	1.80	7.91	1.44		
	Total	8.32	1.83	8.41	1.89	8.11	1.64	0.778	0.438
	*t*	−0.397		0.264		−1.358			
	*p*	0.693		0.793		0.195			

Abbreviations: ILMSC, the distance from the inner of lateral mass to the spinal cord; LMH, the lateral mass height along the C1ZAS trajectory; PSL, the screw length of C1 pedicle screw; RD, the distances from the recess to the dura; RSC, the distance from the recess to the spinal cord; RTF, the distance from the recess to the transverse foramen; TSZ, the tricortical screw zone; ZSL, the screw length of C1ZAS.

### 3.2 Radiographic Parameters

The average RTF, TSZ, LMH, ZSL, PSL, RD, RSC, and ILMSC were 7.71, 6.14, 8.32, 33.23, 26.77, 4.68, 10.02, and 2.91 mm, respectively. The average ideal angled cephalad of C1ZAS was 8.7° ± 3.5°(range 5.2°–12.2°). TSZ was greater than RD but lower than RSC. The medial safe zone of C1ZAS was defined as 4.68 ± 1.42 mm and the inner limited zone was defined as 6.14 ± 1.31 mm. The lateral safe zone of C1ZAS was 7.71 ± 1.42 mm. The average TSZ and ZSL for males were 6.31 and 33.75 mm, respectively, which were greater than those for females (5.62 and 31.76 mm, respectively) (*t* = 2.777, *p* = 0.007; *t* = 4.310, *p* = 0.000). The ZSL was significantly higher than the PSL (*t* = 23.519, *p* = 0.000). The entry point of the C1ZAS was defined as the projection point of the inner of the recess to the posterior arch at least 3 mm below the superior rim of the C1 lamina, and the trajectory was angled cephalad by 8.7° and medially by 0°. The 61 patients (122 sides) with AAD or AAI were classified into three groups: the low‐risk (76 sides, 62%), the intermedial‐risk (18 sides, 15%), and the high‐risk (28 sides, 23%) groups. Moreover, the results of the consistent test analysis showed the ICC of the C1ZAS measurements were 0.930–0.977, which indicated a high consistency of these measurements (Table 2).

**TABLE 2:** Interobserver reliability of radiographic parameter.

	ICC	95% CI	*p*
RTF	0.970	(0.958, 0.979)	< 0.001
RD	0.974	(0.963, 0.982)	< 0.001
RSC	0.966	(0.952, 0.976)	< 0.001
ILMSC	0.948	(0.926, 0.963)	< 0.001
TSZ	0.977	(0.968, 0.984)	< 0.001
ZSL	0.968	(0.955, 0.978)	< 0.001
PSL	0.930	(0.902, 0.951)	< 0.001
LMH	0.960	(0.943, 0.972)	< 0.001

Abbreviations: ILMSC, the distance from the inner of lateral mass to the spinal cord; LMH, the lateral mass height along the C1ZAS traddjectory; PSL, the screw length of C1 pedicle screw; RD, the distances from the recess to the dura; RSC, the distance from the recess to the spinal cord; RTF, the distance from the recess to the transverse foramen; TSZ, the tricortical screw zone; ZSL, the screw length of C1ZAS.

### 3.3 Clinical Outcomes

**FIGURE 5:**
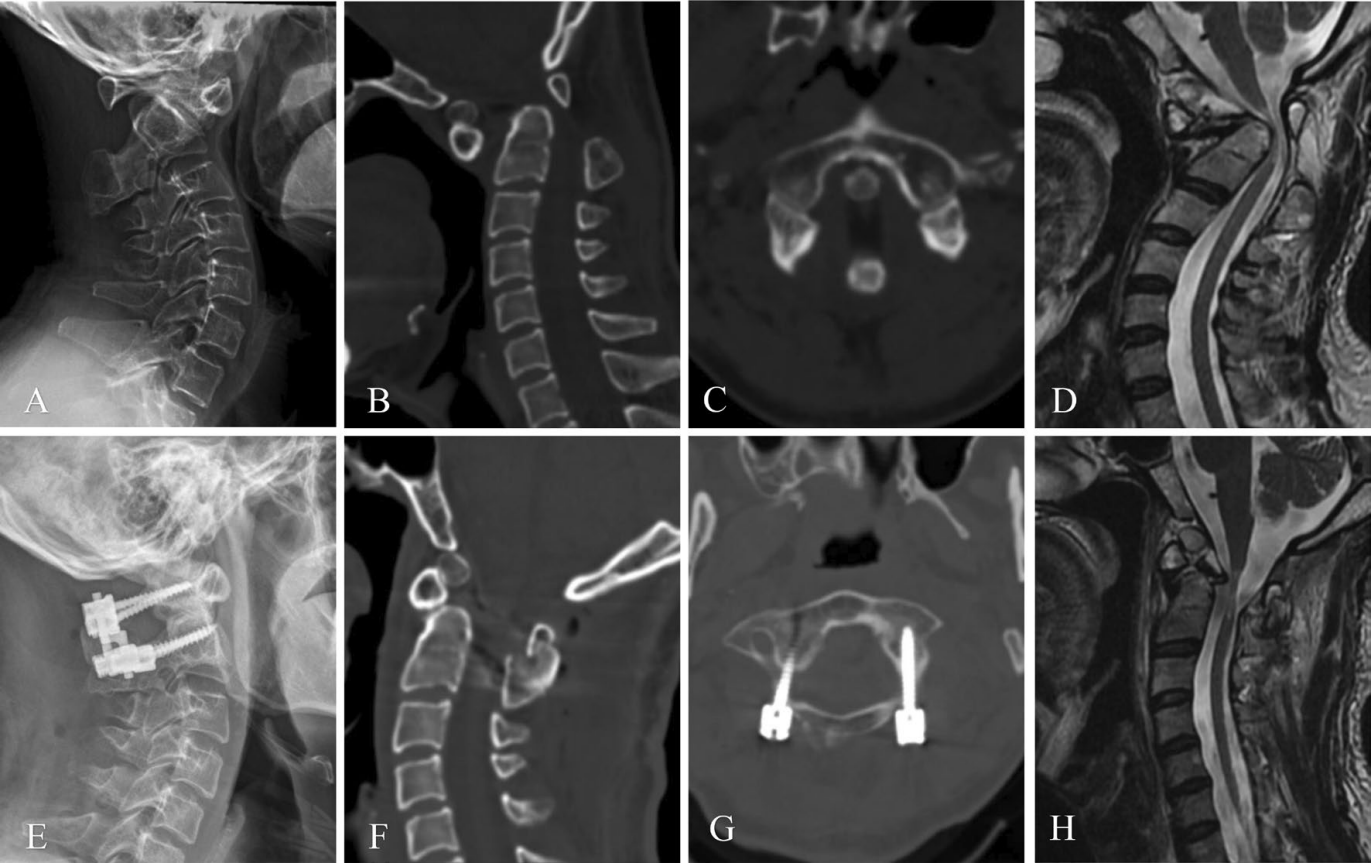
A 39‐year‐old male patient with AAD and left narrow pedicle of atlas underwent treatment with posterior unilateral (left side) C1ZAS with “in–out–in” technique combined with C2 bilateral pedicle screw fixation. (A) The lateral plain radiograph showed AAD. (B,C) Preoperative axial and sagittal CT scans showed AAD combined with Os odontoideum. (D) Preoperative sagittal T2‐weighted MRI showed decompression of the medullary and degenerative changes in C1–C2 level. (E,F) Postoperative lateral plain radiograph and lateral CT showed good reduction and position of the screws. (G) Postoperative axial plane demonstrated successful placement of the left side C1ZAS with “in–out–in” technique, without violation of the TF or dural sac. (H) Postoperative sagittal T2‐weighted MRI showed satisfactory decompression of the SC.

The demographic and clinical data of the patients were summarized in Table 3. C1ZAS were implanted unilaterally in four patients and bilaterally in another four patients. The clinical symptoms and signs of all eight patients improved significantly 3 days after surgery. Postoperative CT and MRI demonstrated that satisfactory C1ZAS placement was achieved in all patients using the “in–out–in” technique, with no instances of implant failure (Figure 5). No TF violation or VA/dura injury was noted in all patients. The average time to fusion was 4 months (range: 3–6 months). The mean JOA score improved from 13.4 ± 2.6 to 15.6 ± 1.8 3 months postoperatively in the eight cases where it was measured (*t* = −4.277, *p* = 0.004).

**TABLE 3:** Demographic and clinical data of the AAD patients with C1ZAS placement.

No.	Sex	Age (year)	Diagnosis	Clinical symptoms	Radiological anomalies	Narrow C1 pedicle	Preoperative ADI (mm)	Postoperative ADI (mm)	C1ZAS with “in–out–in” technique
1	M	64	AAD	Neck pain, chest, abdomen, and extremity numbness	—	Bilateral side	6.06	1.41	Bilateral side
2	F	70	AAD	Dizziness and extremity numbness	Os odontoideum	Bilateral side	6.48	1.99	Bilateral side
3	M	11	AAD	Torticollis and extremity weakness	Os odontoideum	Bilateral side	8.68	2.25	Bilateral side
4	F	59	AAD	Neck discomfort and dizziness	Os odontoideum	Left side	3.42	1.53	Bilateral side
5	F	40	AAD	Neck discomfort, upper limb numbness, and dizziness	Os odontoideum	Right side	7.15	1.01	Right side
6	F	57	AAD	Cervical movement limitation and right upper limb numbness	—	Left side	6.34	2.29	Left side
7	M	39	AAD	Cervical movement limitation and both lower limbs numbness	Os odontoideum	Left side	10.34	2.07	Left side
8	M	69	AAD	Upper limbs numbness and weakness	—	Left side	7.19	1.5	Left side

## 4 Discussion

### 4.1 Main Results and Clinical Significance

In this study, we proposed and validated the feasibility and safety of the C1ZAS with “in–out–in” technique via imaging evaluation and the preliminary clinical application. This technique had a trajectory parallel to the sagittal plane, penetrating the recess of the atlas and providing tricortical or quadricortical purchase for rigid fixation. Moving the entry point and trajectory inward and away from the TF, the ZSL is significantly higher than the PSL. This approach offered superior pullout strength compared with C1 PS/LMS, reduced the risk of C2 nerve root irritation, and minimizes bleeding from the venous plexus [3, 9, 10]. Base on the measurements of RTF, RD, RSC, ILMSC, and LMH, the C1ZAS with “in–out–in” technique had enough space to be placed into the atlas and avoids VA/dura injury. Then we defined the TSZ as the safe area in which the C1ZAS with “in–out–in” technique was placed and it was safe to the breach the medial wall of the pedicle up to 50% of the screw diameter [11, 12, 13]. In addition, this parameter possessed excellent reproducibility regardless of the working experience of the observers (Table 2). We reviewed the feasibility and safety of C1ZAS with “in–out–in” technique in eight patients. Despite the small sample size, our study demonstrated favorable results for the C1ZAS with “in–out–in” technique in patients with AAD and unilateral/bilateral narrow C1 pedicles.

### 4.2 Biomechanical Stability of C1ZAS With “In–Out–In” Technique

Regardless of the type of atlantoaxial surgery, the success of the operation relied on good reduction, decompression of the SC, reliable fixation, and bone fusion. In cases of failed C1 PS placement, C1 posterior arch crossing screws or C1 laminar hooks were used as salvage fixation for AAD [14, 15, 16, 17]. These clinical results confirmed that fixation with C1 posterior arch screws or C1 laminar hooks was safe, providing good biomechanical stability, and improving neurological function of patients. However, these techniques do not offer rigid fixation of the atlas, particularly in cases of irreducible AAD [18, 19]. Meanwhile, as the atlas must be pulled and reduced in AAD, insufficient screw holding force can result in screws being pulled out during the operation or loosening before bone fusion. Furthermore, achieving firm internal fixation in revision surgery may be challenging due to the prior disruption of the atlantoaxial anatomical structure and the potential presence of associated malformations [20]. The “in–out–in” technique was first used for thoracic fixation as a salvage technique for the PS placement in the thoracic dysplastic pedicle [21]. This technique allows for tricortical or quadricortical fixation, providing rigid stability. Theoretically, this results in greater biomechanical stability compared with other salvage techniques [22].

### 4.3 Risk Assessment of C1ZAS Placement With “In–Out–In” Technique

In the process of C1 PS placement, the risk of VA injury is a non‐negligible factor. The shortest distance between the VA and VA groove tended to increase from the outer aspect to the inner aspect of the VA groove, as the VA progressively runed anteromedially and superiorly into the spinal canal [23]. The shortest distance between the VA and VA groove measured 1.5 ± 0.7 mm at the C1 PS entry point, while it was 2.3 ± 1.1 mm at the entry point in C1ZAS. The risk of VA injury is lower with C1ZAS compared with C1 PS in patients with unilateral/bilateral narrow C1 pedicles.

Moving the entry point and trajectory inward away from the TF can prevent VA injury. Furthermore, the rotation of the atlas during drilling, tapping, or screw insertion is also a crucial factor contributing to VA injury [24, 25, 26]. A previous study involving 25 cases of screws breaching the pedicle cortex found that 84% of these breaches deviated laterally, violating the TF rather than the vertebral canal [27]. Forceful unilateral retraction of the muscles necessary to medially angulate the drill or unilateral pressure on C1 by surgical instruments may cause axial rotation of the atlas, thereby decreasing the degree of medial angulation during drilling, tapping, and screw placement. C1 is even more prone to axial rotation compared with C2 due to its natural motion and the absence of a bony block that restricts its rotation [24]. The trajectory of the C1ZAS should be angled cephalad by 8.7° and medially by 0°, which effectively reduce axial rotation of the atlas during drilling, tapping, and screw placement. Adequate preoperative evaluation of the screw trajectory for each patient is essential in CT and MRI. Lin et al. indicated that there is a space medially adjacent to the C1 pedicle, averaging 3.5 ± 0.8 mm between the C1 pedicle and the dura and certain osseous breaches of the medial of C1 pedicle may be easily tolerated and acceptable up to approximately the diameter of a 3.5 mm screw [6]. All C1ZAS were safely inserted in 8 patients with AAD and unilateral/bilateral narrow C1 pedicles and without TF violation or dural laceration. Furthermore, the use of computer‐assisted navigation systems can increase the accuracy and safety of C1ZAS placement [28].

### 4.4 Strengths and Limitations

This study combined the radiographic with clinical outcomes to verify the feasibility and safety of C1ZAS placement with “in–out–in” technique. This technique has been preliminarily demonstrated for screw placement as an alternative option for C1 PS in AAD patients with the C1 pedicle height < 4.0 mm. However, there are still some limitations in the present research. First, this was a preliminary technique report with few cases; therefore, the generalizability of the measured parameters may be limited. Second, this was a retrospective single‐center study and further multicenter studies with large samples and long‐term follow‐up are needed to validate the outcomes. Thirdly, the parameters of MRI in this study may not be accurate to the bone cortex because of the poor imaging of MRI compared with CT. Furthermore, while this technique is clinically feasible, the risk of the dura or the SC violation cannot be completely eliminated. In addition, although this study showed significant clinical and radiological improvements, formal biomechanical evaluation is necessary to clarify the clinical effectiveness and safety of C1ZAS placement with “in–out–in” technique.

### 4.5 Prospects of Clinical Application

C1ZAS placement with “in–out–in” technique showed favorable safety and clinical outcomes as an alternative option for C1 PS in the preliminary clinical application. Base on the results of imaging evaluation and the preliminary clinical application in our study, this technique has the potential to be safe and effective manner for the irreducible AAD and revision surgery. Moreover, formal biomechanical evaluation and multicenter studies with large samples and long‐term follow‐up are necessary to clarify the clinical effectiveness and safety.

## 5 Conclusion

The C1ZAS placement with “in–out–in” technique as an alternative option for C1 PS in AAD patients with the C1 pedicle height < 4.0 mm can achieve tricortical or quadricortical purchase for rigid fixation of atlas. Imaging evaluation and the preliminary clinical application demonstrated the feasibility and safety of C1ZAS. Base on the result of RD in our research, the majority of individuals are suitable for C1ZAS placement with “in–out–in” technique (62% in the low‐risk group). The C1ZAS placement with “in–out–in” technique can be considered as an efficient alternative option when the placement of C1 PS is prohibited due to anatomical constraints.

## Author Contributions

**Zexing Chen:** data analysis and writing. **Xinzhao Huang:** formal analysis and writing. **Xiaobao Zou:** data curation and software. **Peirong Lian:** data curation and software. **Guoqiang Liu:** visualization. **Junlin Chen:** visualization. **Changrong Zhu:** project administration and funding acquisition. **Xiangyang Ma:** project administration and funding acquisition.

## Conflicts of Interest

The authors declare no conflicts of interest.
